# Urolithiasis: Update on Metabolic Evaluation of Stone Formers

**DOI:** 10.1100/tsw.2005.118

**Published:** 2005-11-11

**Authors:** Sangtae Park, Margaret S. Pearle

**Affiliations:** Department of Urology, The University of Texas Southwestern Medical Center, Dallas, Texas, USA

**Keywords:** nephrolithiasis, metabolic evaluation, mineral metabolism

## Abstract

Metabolic abnormalities are identified in over 90% of stone formers and the institution of preventative dietary and medical measures has resulted in substantial reduction in stone recurrence rates. We review the contemporary approach to metabolic evaluation of urolithiasis.

A careful medical and dietary history, stone analysis, serologic tests, and urinalysis constitute the initial screening regimen in patients who have been diagnosed with stones. Risk stratification of patients, based on the outcome of the initial screening tests, determines the need for and extent of urinary evaluation in individual stone formers. Conservative dietary measures or a simple metabolic evaluation and treatment has been described for first-time or low-risk stone formers, although the number of 24-h urine collections needed is debatable. A more extensive metabolic evaluation is recommended for recurrent or high-risk stone formers or for those in whom empiric treatment or medical therapy based on simplified evaluation is unsuccessful.

Regardless of etiology, all stone formers should be counseled on dietary measures for stone prevention. The need for medication is determined by the results of 24-h urine analysis and the risk level of the patient. Cost effectiveness of the metabolic evaluation and treatment is strongly influenced by recurrence rate and efficacy of therapy.

Metabolic evaluation and treatment has clearly been shown in randomized trials to reduce stone recurrence rates. Further study will determine the extent of evaluation necessary and the need for selective vs. empiric medical therapy for first-time and recurrent stone formers.

### INTRODUCTION

The prevalence of nephrolithiasis in the U.S. is estimated at 5–12%; for a 70-year-old man, the lifetime chance of being diagnosed with a stone is nearly 1 in 8[1,2,3]. As such, upper urinary tract stones are responsible for significant morbidity, loss of work, and medical cost. In the U.S., estimates of the annual medical expenditure for a diagnosis of nephrolithiasis approaches $2.1 billion, without taking into account lost wages and reduced work productivity[4]. Beyond the immediate effects of an acute stone event, long-term effects such as loss of renal function are rarely taken into account in cost estimates of stone disease. Indeed, a French study of over 1300 new cases of end-stage renal disease requiring dialysis found that in 3.2% cases renal failure was a direct result of stone disease[5].

In addition to the morbidity of an acute stone event, upwards of 50% of patients with symptomatic upper tract stones ultimately require surgical intervention[6,7]. As such, the goal of medical treatment is to prevent disease progression and recurrence, and potentially to reduce stone burden[8]. Indeed, after experiencing an initial episode of renal colic, most patients are receptive, at least in the short term, to preventive measures recommended by their physicians[9].

In light of these goals, a variety of environmental and genetic risk factors have been identified that may be targeted for medical intervention. There are a variety of approaches to the metabolic evaluation of stone formers. Although there is no uniformity in the literature regarding the optimal evaluation and treatment program, several European consensus conference statements have been published on this subject[10,11,12]. In this article, we discuss our approach to the metabolic evaluation of stone disease and summarize the evidence in support of its use.

### DIAGNOSTIC CLASSIFICATION

Calcium-containing stones (calcium oxalate, calcium apatite, and brushite) represent approximately 75% of upper tract stones and the remaining 25% are comprised of struvite, cystine, uric acid, and other stones. Determination of stone composition is usually performed using X-ray crystallography or infrared spectrophotometry[13], although a relatively new technique using coherent scatter analysis has been described that determines stone composition *in vivo*[14].

Table 1 summarizes the diagnostic classification of nephrolithiasis, including the prevalence and corresponding metabolic or environmental risk factors for each diagnostic category. Some investigators have suggested that comprehensive metabolic testing can be foregone in patients with noncalcium-containing stones such as uric acid and cystine, in whom the underlying pathophysiologic disorder can be diagnosed with good reliability simply by knowing the composition of the stone[13,15]. For example, cystine stones are diagnostic for cystinuria, uric acid stones imply acidic urine, and struvite stones are associated with recurrent urinary infection with urease-producing bacteria[16].

#### Calcium-Containing Stones

##### *Hypercalciuria*

Among calcium-containing stones, calcium oxalate occurs most frequently (60% of all stones), followed by hydroxyapatite (20%) and brushite (2%). Hypercalciuria is classified as absorptive, renal, or resorptive based on the underlying pathophysiologic abnormality leading to hypercalciuria, and treatment is ideally directed at correcting the underlying metabolic derangement.

Absorptive hypercalciuria (AH) is due to intestinal overabsorption of calcium leading to transient elevation of serum calcium, increased filtered load of calcium, and suppressed serum parathyroid hormone (PTH), with subsequent increased renal calcium excretion. AH is defined by increased urinary calcium excretion after an oral calcium load (>0.2 mg/mg creatinine) and is classified as Type II when urinary calcium normalizes with dietary calcium restriction and Type I when it is diet unresponsive.

In renal hypercalciuria, impaired renal tubular reabsorption of calcium results in elevated urinary calcium levels, transient decreased serum calcium, and a secondary increase in serum PTH. Resorptive hypercalciuria is an uncommon abnormality that is most commonly associated with primary hyperparathyroidism. Excessive PTH secretion from a parathyroid adenoma leads to bone resorption and hypercalcemia as well to increased renal synthesis of 1,25 $(OH)_2$ vitamin D, which in turn enhances intestinal absorption of calcium. Although most patients demonstrate hypercalcemia and hypercalciuria, in some cases, normal serum calcium is associated with an inappropriately high serum PTH.

**TABLE 1**
**Classification of Nephrolithiasis**

| Condition | Metabolic/Environmental Defect | Prevalence |
|---|---|---|
| Hypercalciuria | | |
| Absorptive hypercalciuria | Increased GI calcium absorption | 20–40% |
| Renal hypercalciuria | Impaired renal calcium reabsorption | 5–8% |
| Resorptive hypercalciuria | Primary hyperparathyroidism | 3–5% |
| Hyperuricosuric calcium nephrolithiasis | Dietary purine excess, uric acid overproduction or overexcretion | 10–40% |
| Hypocitraturic calcium nephrolithiasis | | |
| Chronic diarrheal syndrome | GI alkali loss | 10–50% |
| Distal RTA | Impaired renal tubular acid excretion | |
| Thiazide-induced | Hypokalemia and intracellular acidosis | |
| Hyperoxaluric calcium nephrolithiasis | | |
| Primary hyperoxaluria | Genetic oxalate overproduction | 2–15% |
| Dietary hyperoxaluria | Excessive dietary intake | |
| Enteric hyperoxaluria | Increased GI oxalate absorption | |
| Gouty diathesis | Low urine pH | 15–30% |
| Cystinuria | Impaired renal cystine reabsorption | <1% |
| Infection stones | Urinary infection with urease-producing bacteria | 1–5% |

### *Hyperuricosuria*

Hyperuricosuria, defined as urinary uric acid >600 mg daily, is present in up to 10% of calcium stone formers[17]. Hyperuricosuria predisposes to calcium or uric acid stone formation by causing supersaturation of the urine with respect to monosodium urate. At pH <5.5, the undissociated form of uric acid predominates, leading to uric acid stone formation. At pH >5.5, sodium urate formation promotes calcium oxalate stone formation through heterologous nucleation[18]. The most common cause of hyperuricosuria is increased dietary purine intake since uric acid represents the end product of purine metabolism. However, acquired and hereditary diseases, such as gout and hematologic disorders, may be accompanied by hyperuricosuria.

### *Hypocitraturia*

Hypocitraturia is defined as urinary citrate <320 mg daily. Citrate inhibits calcium stone formation by complexing with calcium and reducing urinary saturation of calcium salts and by directly preventing spontaneous nucleation of calcium oxalate[19].

Citrate excretion is determined primarily by the acid-base state. In states of metabolic acidosis, urinary citrate is reduced as a result of enhanced renal tubular reabsorption and decreased synthesis of citrate in peritubular cells[20]. Distal renal tubular acidosis, a defect in distal renal tubular urinary acidification, is characterized by high urine pH, hypocitraturia, and low serum potassium and bicarbonate. Chronic diarrheal states produce hypocitraturia by causing systemic acidosis as a result of intestinal alkali loss in the stool. Thiazide diuretics are associated with hypocitraturia by inducing hypokalemia and intracellular acidosis. Excessive animal protein provides an acid load leading to hypocitraturia. Lastly, lactic acidosis during strenuous exercise can cause hypocitraturia.

#### *Hyperoxaluria*

Hyperoxaluria (urinary oxalate >40 mg/day) leads to increased urinary saturation and crystallization of calcium oxalate. Hyperoxaluria may be inherited or acquired. Primary hyperoxaluria results from a rare autosomal recessive disorder in glyoxylate metabolism, leading to enhanced conversion of glyoxylate to oxalate and markedly high urinary levels of oxalate ensue (>100 mg/day). Systemic oxalosis and severe stone formation occur, and without treatment, end-stage renal failure is inevitable[21].

The most common cause of acquired hyperoxaluria is enteric hyperoxaluria, typically associated with chronic diarrheal states. Fat malabsorption results in complexation of fatty acids with calcium, thereby reducing calcium oxalate complexation and increasing oxalate reabsorption[22].

Hyperoxaluria in the absence of intestinal disease is commonly due to increased dietary intake of oxalate-rich foods such as nuts, chocolate, brewed tea, spinach, and broccoli. In addition, severe calcium restriction reduces intestinal binding of oxalate and increases intestinal oxalate absorption.

#### *Low Urine pH*

The undissociated form of uric acid predominates at low urine pH (<5.5), leading to uric acid or calcium stone formation. Calcium oxalate stones form as a result of heterologous nucleation with uric acid crystals. Although any disorder leading to low urine pH may predispose to stone formation, “gouty diathesis” refers to a stone-forming propensity characterized by low urine pH of unclear etiology with or without associated gouty arthritis[23].

### Noncalcium-Containing Stones

#### *Uric Acid Stones*

The three main determinants of uric acid stone formation are low pH, low urine volume, and hyperuricosuria, although low urine pH is the most important pathogenetic factor. The etiology of low urine pH in uric acid stone formers has not been fully elucidated. However an association between uric acid stone formation and noninsulin-dependent diabetes has recently been reported[24]. Insulin resistance, the cause of noninsulin diabetes, has been shown to impair renal ammoniagenesis, which in turn reduces urinary ammonium and lowers urine pH.

#### *Cystine Stones*

Cystinuria is an autosomal recessive disorder characterized by a defect in intestinal and renal tubular transport of dibasic amino acids, resulting in excessive urinary excretion of cystine[25]. Cystine is poorly soluble in urine and, therefore, precipitation of cystine and subsequent stone formation occur at physiologic urine conditions.

#### *Infection Stones*

Struvite stones (magnesium ammonium phosphate) occur only in association with urinary infection by urea-splitting bacteria. Under these conditions, urinary urea is hydrolyzed to ammonia by bacterial urease, resulting in alkaline urine that further promotes phosphate dissociation and allows formation of magnesium ammonium phosphate stones[16].

## MEDICAL EVALUATION

### History

The initial step in the metabolic evaluation of stone formers is a thorough medical and dietary history. Details of the history allow risk stratification of stone-forming patients, which can be used to ascertain the extent of evaluation needed.

A careful history should elicit a previous personal or family history of stone disease. The composition of previous stones and the type and success of previous surgical intervention should also be sought. If previous 24-h urine analyses were obtained, the levels of stone risk factors should be reviewed if available. Dietary habits and fluid intake are assessed for evidence of environmental risk factors. A low fluid intake has been shown in a randomized trial to be associated with an increased risk of stone recurrence[26,27]. Excessive animal protein intake has been associated with hyperuricosuria, decreased urinary pH, and hypocitraturia[28,29,30,31]. Excessive dairy and vitamin D intake may lead to hypercalciuria, although the role of dietary calcium in stone formation is controversial[32,33,34,35,36]. High doses of vitamin C (>2 g daily) may lead to hyperoxaluria[37].

The medical history may identify underlying risk factors predisposing to stone disease. Frequent urinary tract infections may suggest struvite stones[16]. A history of systemic illnesses such as chronic diarrhea[38], hyperthyroidism, inflammatory bowel disease[38], primary hyperparathyroidism[39], renal tubular acidosis[40,41], sarcoidosis[39], gout[42], and malignancy may be associated with an increased risk of stone formation. The metabolic syndrome, characterized by dyslipidemia, insulin resistance, elevated blood pressure, and abdominal obesity, has recently been shown to be associated with uric acid stones as a result of impaired renal ammoniagenesis[43].

Previous surgery, including intestinal bypass or bowel resection[38] and thyroid or parathyroid surgery, should be elicited because of the association of calcium stones with malabsorptive states or with primary hyperparathyroidism. Previous urinary tract surgery may lead to obstruction or stasis of the collecting system, predisposing to stone formation[44].

Medications may constitute a direct or indirect cause of stone disease. Corticosteroids increase intestinal calcium absorption, leading to hypercalciuria and increased saturation of calcium-forming salts. Hypercalciuria can also occur with ingestion of aluminum-containing antacids that bind phosphate and increase intestinal calcium absorption[45] with loop diuretics that reduce renal calcium reabsorption and with vitamin D that enhances intestinal calcium absorption. Colchicine, probenecid, and chemotherapeutic agents can cause hyperuricosuria by increasing uric acid production or enhancing uric acid excretion. Thiazide diuretics cause hypokalemia and intracellular acidosis, leading to hypocitraturia. As citrate is a potent inhibitor of calcium stone disease, hypocitraturia leads to reduced inhibitory activity and an increased risk of stone formation. Acetazolamide, a carbonic anhydrase inhibitor, leads to urinary alkalinization and hypocitraturia. Triamterine, indinavir[46], ephedrine, and guaifenesin[47] are associated with stone formation as a result of direct precipitation of the drug or its metabolites, which are typically poorly soluble in urine.

Genetic forms of stone disease associated with inborn errors of metabolism such as cystinuria[48], primary hyperoxaluria[49], and xanthinuria[50] are often suspected on the basis of family history or early age of onset and diagnosed with laboratory examination or genetic testing. In addition, an increased incidence of stone disease is found in calcium stone formers with a family history of stones. The odds ratio for family history of urolithiasis in stone formers has been estimated at 2 to 3[51,52]. Recently, a gene associated with hypercalciuria was identified that shares homology with the rat soluble adenylate cyclase gene[53,54].

### Laboratory Testing

All patients should undergo a baseline urinalysis, culture, and plain radiograph of the kidneys, ureters, and bladder. A low urine pH (<5.5) on spot urinalysis may suggest gouty diathesis, while a high pH

(>7.5) suggests infection with a urea-splitting organism. Urinary culture demonstrating growth of *Proteus*, *Klebsiella*, *Pseudomonas*, and *Staphylococcus* sp. may suggest struvite stones[16]. Radiographic studies may distinguish radio-opaque stones (calcium oxalate, calcium phosphate, struvite, and cystine) from radiolucent stones such as uric acid.

A stone analysis should be obtained if possible after stone passage or retrieval, as stone composition may reveal the underlying etiology of stone formation such as cystinuria with cystine stones, low urine pH with uric acid stones, and recurrent urinary tract infections with urea-producing bacteria with struvite stones.

Blood work should include serum creatinine, potassium, bicarbonate, calcium, phosphorus, uric acid, and intact parathyroid hormone (iPTH). Serologic findings may also suggest an underlying metabolic cause for stone disease. Primary hyperparathyroidism is associated with elevated serum calcium, low serum phosphorus, and inappropriately high iPTH[55]. Renal tubular acidosis is characterized by low serum bicarbonate, hypokalemia, and hyperchloremia[56]. Hyperuricemia is indicative of gout and poses a risk for uric acid or calcium stones.

The cornerstone of the metabolic evaluation is analysis of a 24-h urine collection for stone risk factors. The 24-h urine analysis aims to identify metabolic, environmental, or physicochemical abnormalities resulting in stone formation and should be delayed until 4–6 weeks after an acute stone when the patient has resumed normal activity and dietary intake[57,58]. Metabolic risk factors include hypercalciuria, hyperoxaluria, hypocitraturia, hyperuricosuria, and low urine pH. Environmental risk factors include low total urine volume, elevated urinary sodium, hypomagnesiuria, and high urine sulfate[59]. Physicochemical risk factors include increased urinary saturation of calcium oxalate, calcium phosphate, uric acid, and monosodium urate.

### Risk Stratification

Stratification of patients into low- and high-risk groups guides the need for and extent of metabolic evaluation, although the ultimate decision to proceed with an evaluation is shared by the physician and patient. Several stratification schemes have been proposed in the literature, including simple vs. complicated stone disease[12], calcareous vs. noncalcareous[15], or first-time low-risk vs. first-time high-risk and recurrent stone formers[60]. In general, patients with bilateral stones, recurrent stones, a family history of stone disease, a solitary kidney, medical risk factors such as primary hyperthyroidism or hyperparathyroidism, distal RTA, gout, bone disease, or bowel disease, and all children, constitute a high-risk group and warrant evaluation. In addition, patients who can ill afford a debilitating acute stone event such as airline pilots, truck drivers, or frequent business travelers should be evaluated. However, patients should be counseled that prospective studies on first-time stone formers have demonstrated recurrence rates ranging from 12–50% at 5 years following diagnosis of the first stone[27,61,62], which may prove reason enough to proceed with evaluation of all stone formers.

#### *First-Time, Low-Risk Stone Formers*

Although first-time stone formers without clear-cut risk factors are considered at low risk of recurrence, Pak demonstrated metabolic abnormalities in nearly 80% of single stone formers and found the same spectrum and frequency of metabolic abnormalities in this patient group as in recurrent stone formers[63]. Yagisawa and associates identified 1.46 abnormalities per patient in first-time stone formers, with low urine volume and absorptive hypercalciuria occurring most frequently[64].

Nevertheless, most urologists in practice do not perform extensive metabolic evaluations on first-time low-risk stone formers. A favorable response to dietary measures alone suggests that such an approach may not be unreasonable. In a prospective randomized trial among first-time idiopathic calcium stone formers, Borghi and colleagues demonstrated that a high fluid intake reduced the rate of stone recurrence from 27 to 12% over 5 years[27]. Kocvara and associates demonstrated a nearly threefold higher stone recurrence rate among patients randomized to general dietary recommendations and limited metabolic screening vs. those randomized to a specific dietary regimen based on a comprehensive metabolic evaluation and close follow-up[65].

Consequently, low-risk first-time stone formers may warrant a trial of conservative dietary measures rather than evaluation and drug therapy. Since drug therapy is not without risk and cost, this strategy deserves consideration from both a cost-effectiveness and morbidity standpoint[66].

#### *First-Time, High-Risk, and Recurrent Stone Formers*

Risk factors for recurrence in first-time stone formers include active medical disease related to urolithiasis; male gender[62]; multiple stones[67]; family history; stone composition of cystine, struvite, or uric acid; and residual stones after surgical treatment[64]. In addition, hyperuricemia, recurrent urinary tract infection, and chronic diarrhea or bowel resection place even first-time stone formers at high risk of recurrence. For these high-risk first-time stone formers, empiric therapy or limited evaluation and treatment is warranted as recurrence rates have been reported to range from 12–50% at 5 years[27,61,62].

Although the need for evaluation of patients at high risk of stone recurrence is generally accepted, the number of 24-h collections necessary is subject to debate. With an extensive evaluation, most recurrent stone formers are found to have at least a single metabolic abnormality, and over half have more than one abnormality[23].

Yagisawa and colleagues compared limited (one or two, 24-h urine collections) and comprehensive (two random 24-h urine collections, one 24-h urine collection after a week of dietary restriction, and a calcium load test) metabolic evaluations in recurrent calcium stone formers and found that the comprehensive approach yielded a specific metabolic abnormality in 90% of patients compared with 68% of patients undergoing a single urine collection and 75% of patients undergoing two 24-h urine collections[68]. The average total number of specific metabolic abnormalities per patient was approximately 50% higher based on a comprehensive metabolic evaluation compared with a single 24-h urine collection (1.59 vs. 0.94, $p < 0.05$). Hypercalciuria, hyperoxaluria, and hypocitraturia were diagnosed significantly more often by the comprehensive than the limited evaluation.

Bek-Jensen and Tiselius found intraindividual variation in all measured parameters when comparing a single vs. multiple urine collections. However, they found that in more than 80% of cases, two 24-h urine samples were sufficient to establish whether a serious metabolic derangement was present[69]. After comparing two 24-h urine collections, Parks and associates concluded that intraindividual variability between collections makes a single 24-h urine collection unreliable[70]. Therefore, these investigators routinely perform up to three 24-h collections as part of their comprehensive metabolic evaluation.

In contrast, Pak and coworkers compared two random 24-h urine samples and found a highly significant positive correlation between risk factors in the two samples[71]. Therefore, they concluded that a single urine collection is representative of the primary underlying problems and they proposed a simplified metabolic evaluation consisting of one 24-h sample before and one after dietary modification.

#### Simplified Diagnostic Protocol

The simplified evaluation was originally described to circumvent the daunting complexity of the selective treatment approach[60]. According to this approach, routine serum biochemistries, stone analysis, urinalysis, urine culture, and abdominal X-ray are obtained. A single 24-h urine is collected while the patient maintains an unrestricted diet. The 24-h urine is analyzed for metabolic factors (calcium, oxalate, uric acid, potassium, citrate, pH) and environmental factors (total volume, sodium, sulfate, phosphorus, magnesium), and physiochemical parameters (including urinary saturation of calcium oxalate, brushite, monosodium urate, and uric acid) are calculated using EQUIL 2[72]. Thereafter, short-term (1 week) dietary modification is imposed based on the results of the first 24-h urine collection:

- Increased fluid intake if urinary volume is less than 2 l
- Sodium restriction if urinary sodium exceeds 200 mEq daily
- Oxalate restriction if urinary oxalate exceeds 40 mg daily
- Calcium restriction if hypercalciuria is identified
- Decreased animal protein if hyperuricosuria with elevated urinary sulfate is identified

A follow-up 24-h urine sample is then repeated, reflecting the dietary changes mentioned above. In this manner, patients can be classified into two diagnostic categories: “uncomplicated” and “other” stone disease (Fig. 1). Uncomplicated stone formers include those with calcium oxalate and calcium phosphate stones who have normal serum calcium and uric acid, and no history of recurrent urinary infections, bowel disease, or marked hyperoxaluria. This group can be further separated into those with normocalciuria and those with hypercalciuria. Normocalciuric stone formers include those with gouty diathesis (idiopathic low urine pH), hyperuricosuric calcium oxalate nephrolithiasis, and hypocitraturic calcium oxalate nephrolithiasis. Hypercalciuric stone formers include those with absorptive hypercalciuria, renal hypercalciuria, and dietary hypercalciuria.

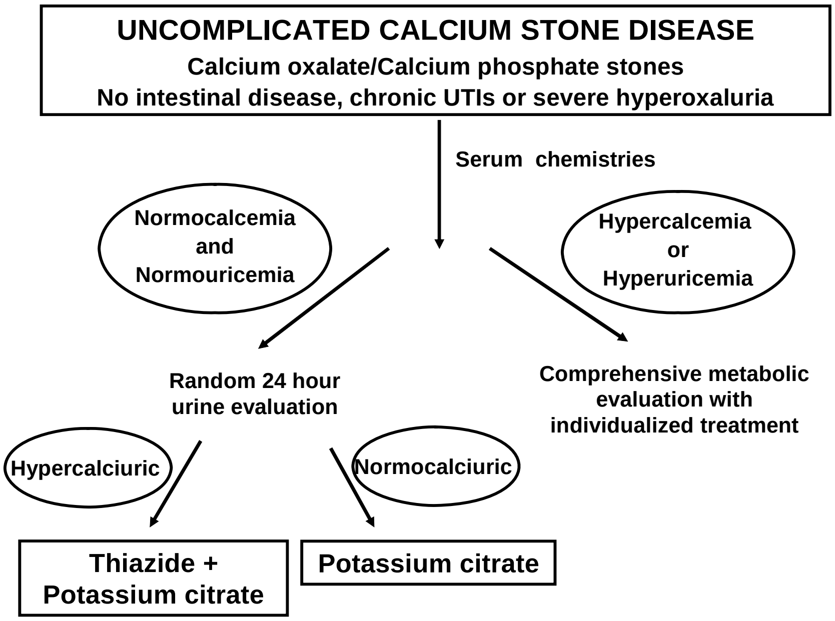

**FIGURE 1.** Algorithm for simplified approach to metabolic evaluation of uncomplicated calcium stone formers.

Stone formers falling into the “other stone disease” group include those with struvite, cystine, uric acid, and medication-related stones and those with primary and enteric hyperoxaluria, primary gout, primary hyperparathyroidism, and renal tubular acidosis.

Based on the results of this simplified protocol, targeted medical therapy can be instituted. For uncomplicated hypercalciuric calcium stone formers, a thiazide diuretic or similar nonthiazide diuretic, Indapamide[73], is prescribed, along with potassium citrate to prevent thiazide-induced hypokalemia or hypocitraturia[74]. For normocalciuric uncomplicated calcium stone formers, potassium citrate is recommended since this drug has been shown to reduce the rate of stone formation in hypocitraturic[75] as well as hyperuricosuric stones formers[76].

For patients producing noncalcium stones, such uric acid, struvite, and cystine stones, specific measures addressing the underlying pathophysiologic problem are initiatied. For instance, urinary alkalinization with potassium citrate or sodium bicarbonate is recommended for patients with low urine pH, antibiotic suppression or a urease inhibitor (acetohydroxamic acid[77,78]) is advised for patients with struvite stones, and alkalinizing agents and/or chelating agents (alpha-mecaptopropionylglycine[79]) are prescribed for patients with cystine stones.

In patients with hypercalcemia, evaluation for primary hyperparathyroidism is undertaken. Those with hyperuricemia and gout are treated with Allopurinol. Lastly, patients with severe hyperoxaluria should be evaluated for primary hyperoxaluria or bowel disease.

#### Comprehensive Metabolic Evaluation

A more extensive evaluation allows careful differentiation of the various pathophysiologic causes of stone disease with the intent of initiating directed medical therapy aimed at correcting the specific underlying metabolic derangement[80]. This approach involves the collection of two 24-h urine specimens while on a random diet. The patient is then instructed to observe a diet restricted in calcium (400 mg/day) and sodium (100 mEq/day) while avoiding oxalate-rich foods. A third 24-h urine specimen is then collected after adhering to the restricted diet for 1 week, after which a fast and load calcium test is performed to differentiate the various subtypes of hypercalciuria. This test involves collecting a 2-h fasting urine specimen that is analyzed for calcium, creatinine, pH, and total volume. Subsequently, a liquid 1-g calcium load is administered, after which a 4-h urine specimen is collected that is analyzed for calcium, creatinine, and total volume. Urinary calcium exceeding 200 mg/day on a restricted diet defines hypercalciuria. Fasting hypercalciuria (urinary calcium/creatinine ratio of 0.11 or greater) with normal serum calcium suggests impairment in renal calcium reabsorption (renal hypercalciuria), but may also be seen in severe forms of absorptive hypercalciuria. Absorptive hypercalciuria is defined by a calcium/creatinine ratio of 0.22 or greater after ingestion of the calcium load. Of note, the fast and load calcium test is only reliable after a week of sodium and calcium restriction.

Treatment is directed at correcting the underlying pathophysiologic abnormality identified during the course of the metabolic evaluation. Hypercalciuria due to intestinal calcium hyperabsorption, renal calcium leak (impaired renal calcium reabsorption), or dietary hypercalciuria (in some cases) is treated with a thiazide diuretic or Indapamide, often along with potassium citrate[73,74,81,82]. Patients with hypocitraturia are typically treated with potassium citrate[75]. Allopurinol is reserved for patients with hyperuricosuria[73], and low urine pH is corrected with potassium citrate.

Although a comprehensive metabolic evaluation makes it possible to differentiate patients in a sophisticated manner, the impact on therapeutic choices may be limited. Consequently, in the current cost-conscious medical setting, several cost analyses have questioned the cost effectiveness of comprehensive metabolic evaluations that were initially proposed over 20 years ago[66]. This approach may be reserved for patients in whom treatment based on a simplified approach is unsuccessful.

### COMPLIANCE WITH METABOLIC EVALUATION AND THERAPY

Overall, the reported patient compliance with physician-recommended work-up for nephrolithiasis has been poor due to a combination of physician- and patient-related factors. Krepinsky and colleagues retrospectively analyzed 97 recurrent stone formers from a Canadian tertiary care setting, of whom 92% had undergone surgical treatment for their stones[6]. Only 56% of physicians were determined to have ordered appropriate testing based on the National Institutes of Health Consensus Conference recommendations, and only 35% of patients were found to have completed the recommended metabolic evaluation[11]. In a similar review of 177 patients in the Netherlands, only 36% of patients remained compliant with the prescribed selective medical therapy at an average follow-up of 5.3 years[83].

Cystinurics are characterized by younger age of onset and markedly higher recurrence rates for stones. Therefore, close follow-up and directed medical therapy are imperative in order to minimize morbidity in this patient population. Review of the medical records of 26 cystinurics managed at a comprehensive kidney stone referral center revealed that after an average follow-up of 38 months, only 4 patients (15%) consistently achieved a target urinary cystine level below 300 mg/l and in 42% of individuals, the target cystine level was never achieved or the patient never returned for follow-up[84]. Suggested reasons for noncompliance were cost of medication and their side effects.

### COST EFFECTIVENESS OF METABOLIC EVALUATION AND TREATMENT

In developed countries, medical costs continue to spiral upwards and urological diseases have contributed to this rise[85]. Accordingly, cost-effectiveness analyses have the potential to impact health policy and treatment. Chandhoke performed an international cost analysis of directed medical therapy for urolithiasis and found that a metabolic evaluation was not cost effective in first-time stone formers[86]. For recurrent formers, cost effectiveness was largely dependent on the rate of stone recurrence and the efficacy of directed medical therapy in preventing stone recurrence, with cost effectiveness reached only when the rate of stone recurrence exceeded a rate of 0.3–4 stones per year.

Lotan and co-workers used a decision tree model to compare the costs of six medical treatment strategies, namely dietary measures alone (conservative), empiric drug treatment, or directed drug therapy based on simple or comprehensive metabolic evaluation. The recurrence rates, sensitivity of medical evaluation, and risk reduction of medical treatments were derived from the literature. They concluded that in first-time stone formers, conservative therapy is cost effective and efficacious. In recurrent stone formers, conservative treatment was also less costly than any of the drug treatments, but was associated with a higher stone recurrence rate (0.3 stones per patient per year)[66,87]. Comprehensive evaluation and directed medical therapy offered no advantage in cost or efficacy over empiric treatment or simplified medical evaluation and treatment. However, with a limited metabolic evaluation and directed drug therapy or empiric drug treatment only, stone recurrences can be prevented at a modest incremental cost in patients at high risk of developing recurrent stones.

### CONCLUSIONS

Metabolic evaluation is an important component of treatment for patients with nephrolithiasis. Although empiric drug therapy may provide effective prophylaxis against stone recurrence[88], treatment based on metabolic evaluation allows classification of patients into simple diagnostic groups to which an uncomplicated treatment algorithm can be applied in a cost-effective manner. Furthermore, evaluation is advantageous because of the additional medical information it provides.

Although debate continues among centers of excellence as to the details and utility of a simplified vs. comprehensive metabolic evaluation, physicians can expect selective medical therapy to achieve a remission rate of over 80%, and an overall reduction in the rate of individual stone formation of nearly 90%[81].
